# HPLC/MS characterization of *Syzygium aromaticum* L. and evaluation of its effects on peritoneal adhesion: Investigating the role of inflammatory cytokines, oxidative factors, and fibrosis and angiogenesis biomarkers

**DOI:** 10.14814/phy2.15584

**Published:** 2023-01-25

**Authors:** Elham Moradi, Hassan Rakhshandeh, Vafa Rahimi Baradaran, Mobarakeh Ghadiri, Maedeh Hasanpour, Mehrdad Iranshahi, Vahid Reza Askari

**Affiliations:** ^1^ Pharmacological Research Center of Medicinal Plants Mashhad University of Medical Sciences Mashhad Iran; ^2^ Department of Cardiovascular Diseases, Faculty of Medicine Mashhad University of Medical Sciences Mashhad Iran; ^3^ Biotechnology Research Center, Pharmaceutical Technology Institute, Mashhad University of Medical Sciences Mashhad Iran; ^4^ International UNESCO Center for Health‐Related Basic Sciences and Human Nutrition Mashhad University of Medical Sciences Mashhad Iran; ^5^ Applied Biomedical Research Center Mashhad University of Medical Sciences Mashhad Iran

**Keywords:** anti‐inflammatory, antioxidant, immunomodulation, peritoneal adhesion, *Syzygium aromaticum* L

## Abstract

The dried flower bud of *Syzygium aromaticum* L. (*S. aromaticum*) (Myrtaceae), cloves, have been used for their analgesic and anti‐inflammatory activities. Peritoneal adhesion (PA) is the most common complication of abdominal and pelvic surgeries, which causes significant adverse effects and severe economic burden. The present study aimed to evaluate the preventive effect of *S.* extract (SAE) on PA formation in a rat model. Male Wistar 8‐week‐old rats were randomly divided into sham, control (received vehicle), and treatment (0.25%, 0.5%, and 1% w/v of SAE) groups. The adhesion and related factors were examined using the Nair scoring system and immunological and biochemical kits for the levels of inflammatory cytokines [interleukin (IL)‐6 and tumor necrosis factor (TNF)‐α], growth factors [transforming growth factor (TGF)‐β1 and vascular endothelial growth factor (VEGF)], oxidative [nitric oxide (NO) and malondialdehyde (MDA)], and anti‐oxidative [glutathione (GSH)] factors. Our results figured out that the adhesion score and IL‐6, TNF‐α, TGF‐β1, VEGF, NO, and MDA levels were significantly increased, but the GSH level was decreased in the control group compared to the sham group (*p* < 0.001–0.05). On the other hand, the 0.25% SAE group had a lower adhesion score, and IL‐6, TNF‐α, TGF‐β1, VEGF, NO, and MDA levels were significantly decreased compared with the vehicle group, and the level of GSH was increased (*p* < 0.001–0.05). SAE could efficiently reduce adhesion score and regulate inflammatory cytokines, oxidative and anti‐oxidative factors, and biomarkers of fibrosis and angiogenesis. Therefore, clove extract can be considered a potential candidate for PA management.

## Graphical abstract

This study showed that intra‐abdominally administration of SAE signiﬁcantly reduced the severity of post‐operative PA formation according to alleviating adhesion scores, inflammatory cytokines, oxidative factors, fibrosis, angiogenesis biomarkers, and stimulating anti‐oxidative factors. Therefore, SAE can be considered a potential and cost‐effective agent for preventing post‐operative adhesions.

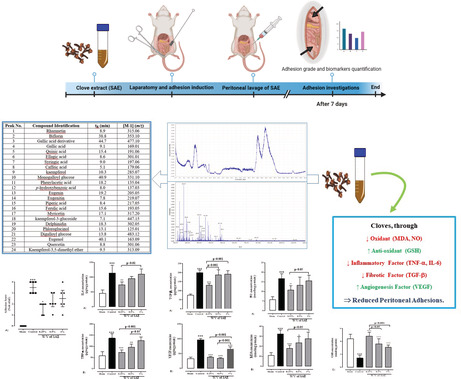

## 1 INTRODUCTION

Peritoneal adhesion (PA) is the most common complication of abdominal and pelvic surgeries, which causes significant economic impacts and considerable morbidities (Arung et al., 2011; Mais, 2014; Van Goor, 2007), including female infertility, small bowel obstructions, and chronic abdominal pain (Arung et al., 2011; Mais, 2014). By definition, PAs are pathological bonds between the omentum, loops of the bowel, and the abdominal wall. These bonds may be classified by origin as innate or acquired, and by their gross features as (1) thin film of connective tissue, (2) thick fibrous bridge containing blood vessels and nerve tissue, or (3) a direct contact between the surface of two organs (Arung et al., 2011; Liakakos et al., 2001). Indeed, PA formation is a part of the innate peritoneal defense mechanism following different trauma to the peritoneum, including surgical handling and instrument contact, foreign materials such as sutures and glove dusting powder, infection, endometriosis, chemotherapy, radiation, malignancy, desiccation, and overheating (Askari et al., 2018; Ghadiri et al., 2021; Jaafari et al., 2021; Rahimi et al., 2017; Rahmanian‐Devin et al., 2021; Roohbakhsh et al., 2020). Notwithstanding approaches to prevent, reduce and treat PA formation can be divided into accurate laparoscopic techniques, using chemical agents, and bio‐absorbable mechanical barriers. In this regard, some methods have been applied, such as physical separation of traumatized serosal areas and intraperitoneal (IP) administration of fibrinolytic, anticoagulant, and anti‐inflammatory agents (Arung et al., 2011; Brochhausen et al., 2012; Mais, 2014; Schnüriger et al., 2011; Wei et al., 2017). Nevertheless, a “gold standard” method to prevent and treat PA formation has not been determined yet (Arung et al., 2011; Brochhausen et al., 2012; Mais, 2014; Schnüriger et al., 2011; Wei et al., 2017).

The exact pathophysiology of PA formation has remained controversial but at the molecular level, including a complex interaction of cytokines, growth factors, and components secreted by platelets, macrophages, and other cells at or near the injured area (Askari et al., 2018; Ghadiri et al., 2021; Jaafari et al., 2021; Rahimi et al., 2017; Rahmanian‐Devin et al., 2021; Roohbakhsh et al., 2020). The balance between fibrin formation and degradation is crucial in determining normal peritoneal healing or adhesion formation. After peritoneal surgery, if incompletely degradation of fibrin and extracellular matrix (by the proenzymes of matrix metalloprotease) does not occur within 5–7 days, it may serve as a scaffold for fibroblasts and growth of new blood vessels to form PAs gradually. Activation of tissue‐type and urokinase‐type plasminogen activators leads to plasmin production and then degradation of fibrin. This process is blocked by plasminogen‐activating inhibitors (PAI)‐1 and 2 (Askari et al., 2018; Ghadiri et al., 2021; Jaafari et al., 2021; Rahimi et al., 2017; Rahmanian‐Devin et al., 2021; Roohbakhsh et al., 2020). After ischemia or inflammation, overexpression of PAI‐1 and PAI‐2 results in PA formation. Inflammatory mediators also play an essential role in PA formation. Specific mediators, such as vascular endothelial growth factor (VEGF), transforming growth factor (TGF)‐β1 and β2, tumor necrosis factor (TNF)‐α, and interleukins (such as IL‐1, IL‐6, and IL‐10) decrease the fibrinolytic capacity of the peritoneum and increase the formation of PA. Oxidative stress and hypoxia due to a further increase in collagen1, fibronectin, tissue inhibitors of metalloproteinases, TGF‐β1, TGF‐β2, IL‐10, and Interferon‐gamma (IFN‐γ) levels in both peritoneal and adhesion fibroblasts, resulting in more formation of PAs (Arung et al., 2011; Cheong et al., 2001; Kamel, 2010; Liakakos et al., 2001; Mais, 2014; Saed et al., 2000; Saed et al., 2001; Schnüriger et al., 2011; Van Goor, 2007).

*Syzygium aromaticum* (L.) Merr. Et Perry (synonym: *Eugenia cariophylata*) is a medium‐sized tree from the *Myrtaceae* family native to the Maluku islands in east Indonesia and cultivated in several parts of the world, including Iran. Cloves are dried flower buds of *Syzygium aromaticum* (L.). This natural product is used as a spice and for traditional medicinal purposes, including dental care, respiratory disorders, allergic disorders, and headaches, because of its antiseptic, analgesic, antiallergic and antioxidant effects. In addition, the clove is one of the richest sources of phenolic and flavonoid compounds. The major component of clove oil is eugenol (72%–90% of the essential oil extract) and lesser amounts of β‐caryophyllene and eugenol acetate (Askari, Rahimi, et al., 2019; Askari & Shafiee‐Nick, 2019; Askari & Shafiee–Nick, 2019; Nourbakhsh & Askari, 2021). The available reports strongly show that clove oil and eugenol and β‐caryophyllene have immunomodulatory, anti‐inflammatory, and anti‐oxidative activities by the probable mechanism of inhibiting lipopolysaccharides (LPS) action and suppression of the nuclear factor‐kB pathway (Abd El Azim et al., 2014; Askari, Rahimi, et al., 2019; Askari & Shafiee‐Nick, 2019; Askari & Shafiee–Nick, 2019; Bachiega et al., 2012; Bhowmik et al., 2012; Chaieb et al., 2007; Cortés‐Rojas et al., 2014; Dibazar et al., 2014; Dibazar et al., 2015; Halder, Mehta, Mediratta, & Sharma, 2011; Nourbakhsh & Askari, 2021; Rodrigues et al., 2009).

Howbeit, to the best of our knowledge, there is no study considering the protective effects of clove on preventing post‐operative PA formation. Therefore, the present study aimed to evaluate the effect of *S. aromaticum* extract (SAE) on this significant complication in a rat model.

## 2 MATERIALS AND METHODS

### 2.1 Drugs and chemicals

Ethanol was purchased from Sigma‐Aldrich Chemical Co.. Ketamine and Xylazine were from ChemiDaru Company (Iran). The injectable normal saline serum was prepared from the Samen pharmacy factory (Iran). IL‐6, TNF‐α, TGF‐β, and VEGF ELISA kits were purchased from IBL‐International Company (Switzerland), and nitric oxide (NO), Malondialdehyde (MDA), and glutathione (GSH) kits were prepared from ZellBio Company (Germany). Other used materials were analytical grade and obtained from Sigma‐Aldrich Chemical Co.

### 2.2 Animals and ethical statement

In this study, 30 healthy male Wistar‐Albino rats (mean weight, 220 ± 20 g and mean age of 8 weeks) without medical interventions history were purchased from the Mashhad University of Medical Sciences animal lab in Mashhad, Iran. They were kept under standard conditions, including separated standard cages and a ventilated room with a 12/12 h natural light–dark cycle, 60 ± 3% humidity, a temperature of 21 ± 2°C, and free access to water and food. With continuous cleaning and daily removal of feces and spilt feeds from cages, proper hygiene was provided. All animals received human care in compliance with institutional guidelines. The ethical committee of Mashhad University of Medical Sciences approved the study protocol by grant number 950312 and the honest number IR.MUMS.MEDICAL.REC.1398.686 (approval date: 2019‐07‐30).

### 2.3 *Syzygium aromaticum* L*.* extract (SAE) preparation

The dried buds of the clove were collected from the local market in Mashhad, Khorasan Razavi, Iran. They were grounded in a milling machine, and for employing the maceration method, 2 L ethanol 70% v/v was added to 150 g clove powder, and the solution was left at 25°C for 48 h with gentle shaking. Then the extract was filtered through filter paper and dried inside the crucible at 45°C. Fresh solutions of 0.25%, 0.5%, and 1% w/v clove extract were made by dissolving 25, 50 and 100 mg of clove extract powder with mixture of NaCl 0.9% w/v and 5% v/v Tween 80 up to 10 ml, respectively (Joseph & Sujatha, 2011; Parsaei et al., 2013).

### 2.4 Liquid chromatography‐mass spectrometry (LC–MS) apparatus

The liquid chromatography‐mass spectrometry (LC–MS) analysis was done utilizing an AB SCIEX QTRAP (Shimadzu) liquid chromatography coupled with a triple quadrupole Mass Spectrometer. Concisely, liquid chromatography (LC) separation was performed on a Supelco C18 (15 mm × 2.1 mm × 3 μm) column, and mass spectrometry (MS) analysis was carried out in the negative mode of ionization. The study was done at a flow rate of 0.2 ml/min, in which the gradient analysis started with 95% of 0.1% aqueous formic acid, isocratic conditions were maintained for 5 min, and then a 15‐min linear gradient to 40% acetonitrile with 0.1% formic acid was applied. Thereafter, from 20 min to 45 min, the acidified acetonitrile was increased to 100%, followed by 10 min of 100% acidified acetonitrile and 5 min at the start conditions to re‐equilibrate the column. The mass spectra were acquired in 100 to 1200 within 60 min of scan time. Mass feature extraction of the acquired LC–MS data and maximum detection of peaks was done applying the *MZmine* analysis software package (version 2.3).

### 2.5 Experimental protocol and the studied groups

**FIGURE 1:**
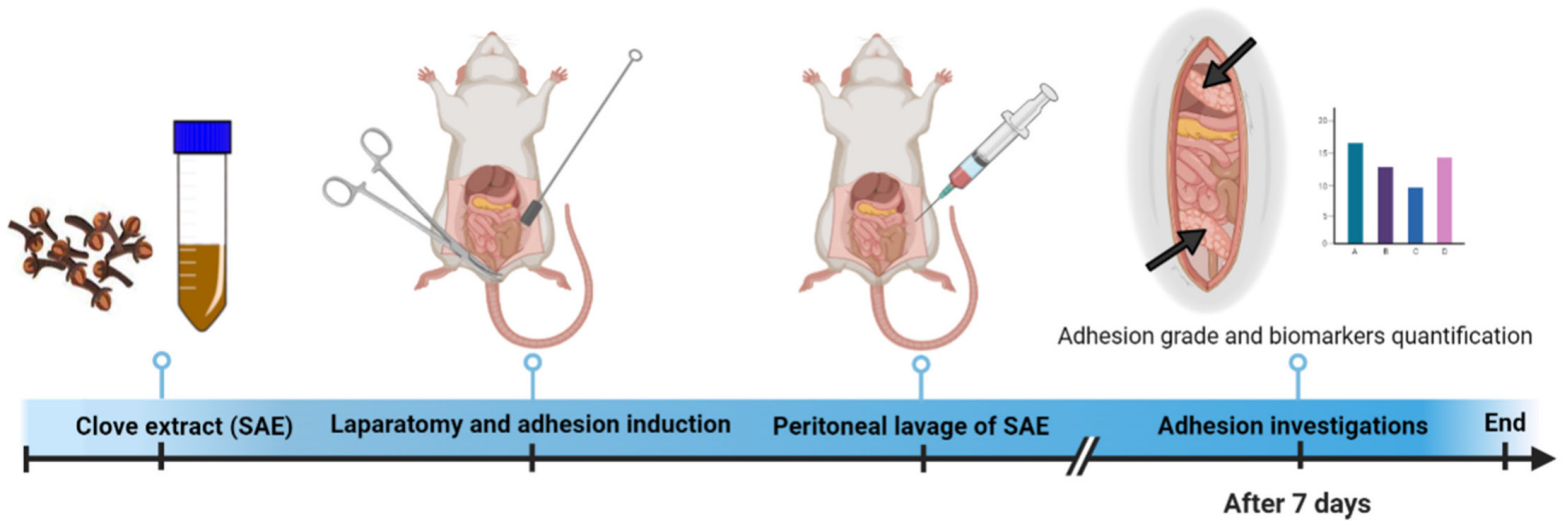
Schematic description of investigation of the protective effects of *Syzygium aromaticum* L. extract on the post‐operative peritoneal adhesion in a rat model.

Thirty healthy Wistar‐Albino male rats were randomly divided into five equal groups (*n* = 6). While rats were anesthetized by IP injection of 10 mg/kg Xylazine HCl and 100 mg/kg Ketamine HCl and 3 mg/kg Acepromazine (Askari et al., 2018; Ghadiri et al., 2021; Jaafari et al., 2021; Rahimi et al., 2017; Rahmanian‐Devin et al., 2021; Roohbakhsh et al., 2020), each rat was laid flat on the back, and abdominal hairs were shaved. The adhesion model was induced by the scraping method (Maghsoudi & Mohammadi, 2009) on the right side of the peritonea. After making lesions and interventions as follows (Figure 1), animals were left in recovery conditions for 7 days (Askari et al., 2018; Ghadiri et al., 2021; Jaafari et al., 2021; Parsaei et al., 2013; Rahimi et al., 2017; Rahmanian‐Devin et al., 2021; Roohbakhsh et al., 2020).

‐Group one: Sham group underwent surgical procedures, but PA was not induced and immediately received the lavage of 2 ml of vehicle (NaCl 0.9% w/v with 5% v/v Tween 80, (Askari et al., 2018; Ghadiri et al., 2021; Jaafari et al., 2021; Rahimi et al., 2017; Rahmanian‐Devin et al., 2021; Roohbakhsh et al., 2020)); −Group two: Control group underwent surgical procedures, and PA was induced and immediately received the lavage of 2 ml of vehicle (NaCl 0.9% w/v with 5% v/v Tween 80, (Askari et al., 2018; Ghadiri et al., 2021; Jaafari et al., 2021; Rahimi et al., 2017; Rahmanian‐Devin et al., 2021; Roohbakhsh et al., 2020)); and ‐Groups three to five: SAE groups underwent surgical procedures, and PA was induced and then immediately received the lavage of 2 ml of 1%, 0.5%, and 0.25% w/v of SAE after PA induction.

### 2.6 Assessment of adhesion grade

On the 7th day of surgery, the laparotomy was done again to examine the severity of the PAs before sampling according to the Nair et al. scoring system (Table 1) by two independent researchers who were blinded to the experimental grouping (Kennedy et al., 1996; Lauder et al., 2011; Nair et al., 1974).

**TABLE 1:** Scoring system for intraperitoneal adhesions according to Nair et al. (Nair et al., 1974)

Score	Description of adhesive bands
0	Complete absence of adhesions
1	Only one band of adhesions among the viscera or between one viscera and the abdominal wall
2	Two bands: among viscera or from viscera to abdominal wall
3	More than two bands: among viscera or from viscera to the abdominal wall or all intestine making a mass without adhesion to the abdominal wall
4	Viscera adhered directly to the abdominal wall, independent of the number and the extension of adhesion bands

### 2.7 Preparation of peritoneal lavage fluid and biomarkers quantification

On the 7th day of surgery, the peritoneal lavage fluid was prepared using 2.5 ml sterilized phosphate‐buffered saline (PBS). In detail, the cecum and the peritoneum area were washed twice, and then the collected fluid was centrifuged at 1008 *g* for 5 min at 4°C. Finally, the supernatant was separated for further investigations. The level of inflammatory (TNF‐α and IL‐6), fibrosis (TGF‐β1) and angiogenesis (VEGF) biomarkers, and oxidative stress indexes (NO, MDA, and GSH) were evaluated in the peritoneal fluid by commercial biochemistry kits according to the manufacturer's instruction (Askari et al., 2018).

### 2.8 Statistical analysis

Data were analyzed using GraphPad Prism software (version 6.01) and represented as mean ± standard deviation (SD) and median ± interquartile range (IQR), according to the nature of parametric or non‐parametric data, respectively. One 1‐way analysis of variance (ANOVA) was performed with Tukey's Kramer multiple comparisons post hoc test for parametric data. In contrast, a Kruskal–Wallis test was performed for the non‐parametric data (adhesion score) with Dunn's multiple comparisons post hoc test (Askari, Baradaran Rahimi, et al., 2019; Rahimi et al., 2018). *p* values ≤0.05 were considered statistically significant (Askari, Rahimi, et al., 2019).

## 3 RESULT AND DISCUSSION

### 3.1 LC–MS analysis of SAE

**FIGURE 2:**
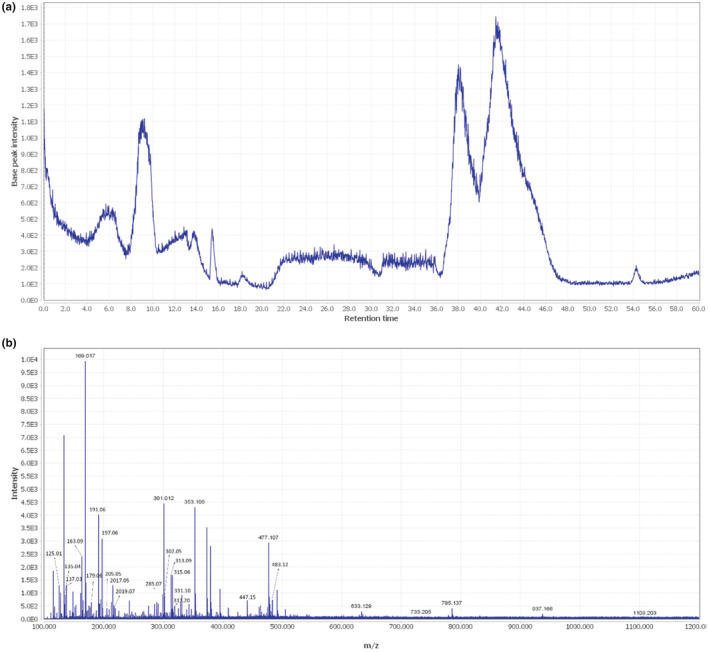
(a) The total ion chromatogram of *Syzygium aromaticum* L. using LC–MS in the negative mode. (b) Mass spectra of *Syzygium aromaticum* L. extract. And their detected compounds that were listed in Table 2.

Twenty‐four compounds were identified in the hydro‐ethanol extract of *Syzygium aromaticum* L., including flavonoids and phenolic acids. Data concerning the identification of the compound are shown in Table 2. The total ion chromatograms of *Syzygium aromaticum* L. extract in ESI− mode is shown in Figure 2a. The MS spectral data were compared with the reported compounds in some previous literature. Figure 2b illustrates the mass spectra of *Syzygium aromaticum* L. extract. Some flavonoids, including rhamnetin, quercetin, kaempferol 3‐glucoside, kaempferol‐3,5‐dimethyl ether, delphinidin, and myricetin were detected in *Syzygium aromaticum* L. extract. Biflorin is a chromone C‐glucoside with a bitter taste and sweet aroma identified in *Syzygium aromaticum* L. extract.

**TABLE 2:** Peak assignment of metabolites in the hydro‐ethanol extract of *Syzygium aromaticum* L. using LC–MS in the negative mode

Peak No.	Compound identification	*t*_R_ (min)	[M‐1] (*m/z*)	Ref.
1	Rhamnetin	8.9	315.06	(Fathoni et al., 2017)
2	Biflorin	38.8	353.10	(Yang et al., 2015)
3	Gallic acid derivative	44.7	477.10	(Faria et al., 2011; Kim et al., 2013)
4	Gallic acid	9.1	169.01	(Faria et al., 2011; Kim et al., 2013)
5	Quinic acid	15.4	191.06	(Fathoni et al., 2017)
6	Ellagic acid	8.6	301.01	(Cortés‐Rojas et al., 2014; Kim et al., 2013)
7	Syringic acid	9.0	197.06	(Jimoh et al., 2017)
8	Caffeic acid	5.1	179.06	(Cortés‐Rojas et al., 2014)
9	kaempferol	10.3	285.07	(Cortés‐Rojas et al., 2014)
10	Monogalloyl glucose	40.9	331.10	(Fathoni et al., 2017)
11	Phenylacetic acid	18.2	135.04	(Jimoh et al., 2017)
12	*p*‐hydroxybenzoic acid	8.0	137.03	(Jimoh et al., 2017)
13	Eugenin	19.2	205.05	(Jimoh et al., 2017; Kaur & Kaushal, 2019)
14	Eugenitin	7.8	219.07	(Jimoh et al., 2017; Kaur & Kaushal, 2019)
15	Piperic acid	8.4	217.05	(Jimoh et al., 2017)
16	Ferulic acid	15.6	193.05	(Cortés‐Rojas et al., 2014)
17	Myricetin	17.1	317.20	(Koba et al., 2011)
18	kaempferol‐3‐glucoside	7.1	447.15	(Jimoh et al., 2017)
19	Delphinidin	18.3	302.05	(Jimoh et al., 2017)
20	Phloroglucinol	13.1	125.01	(Jimoh et al., 2017)
21	Digalloyl glucose	13.8	483.12	(Fathoni et al., 2017)
22	Eugenol	40.1	163.09	(Jimoh et al., 2017; Kaur & Kaushal, 2019)
23	Quercetin	8.8	301.06	(Cortés‐Rojas et al., 2014)
24	Kaempferol‐3,5‐dimethyl ether	9.5	313.09	(Kiran et al., 2021)

### 3.2 The impact of SAE on peritoneal adhesion score

**FIGURE 3:**
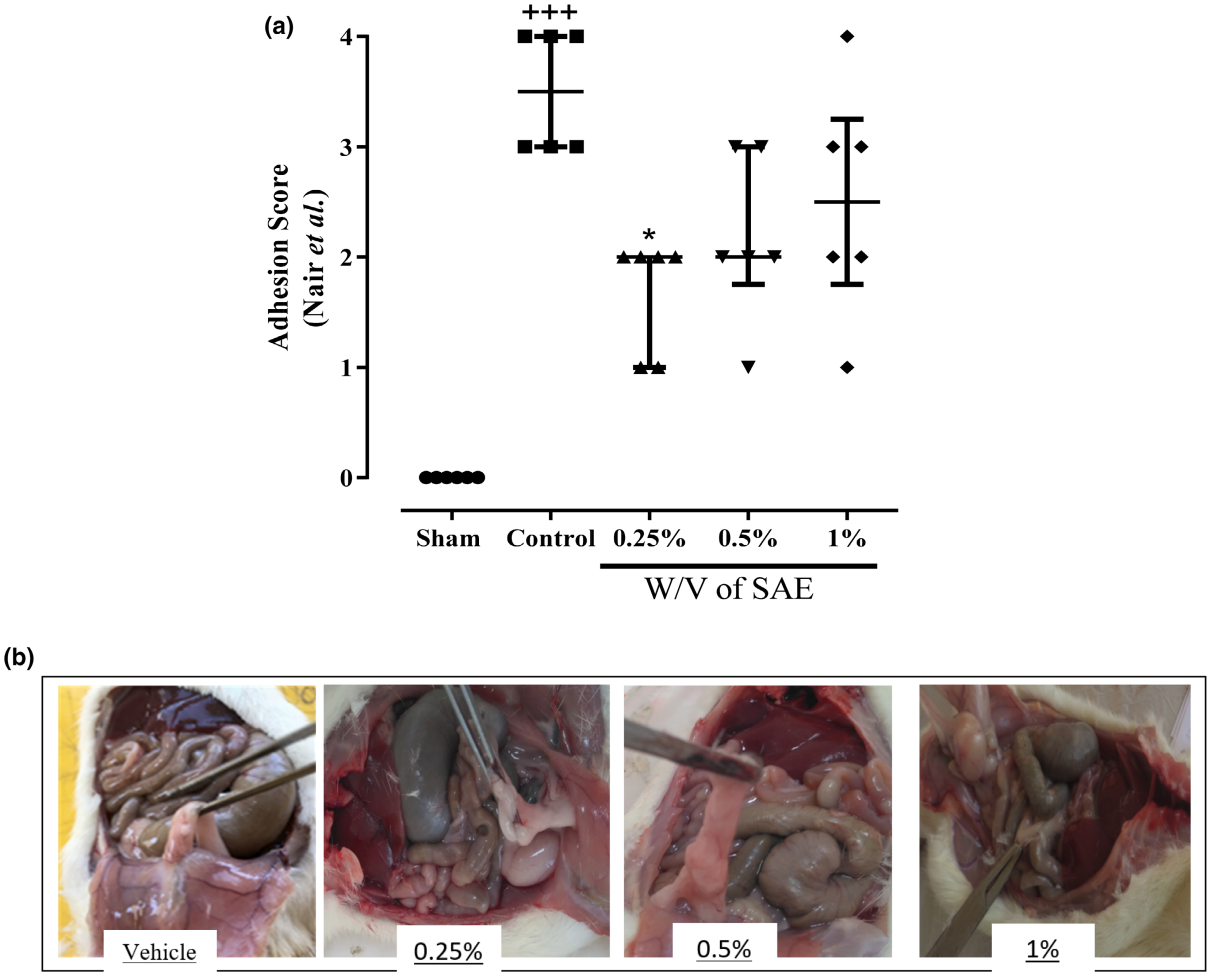
**(**a) The effect of different concentrations of *S. aromaticum* extract (SAE) on adhesion score (Nair et al.) following post‐operational‐induced peritoneal adhesion. b) Shows the images of adhesion bands in different groups. Data were presented as median ± interquartile range (*n* = 6). ^+++^ *p* < 0.001 compared with the sham group and *p < 0.05 compared with the control group.

Our results based on the Nair et al. scoring system (Table 3) represented that control groups had more outstanding adhesion scores in comparison with the shame group (*p* < 0.001, Figure 3a,b). All three concentrations of SAE reduced PA compared with the control group. However, only the 0.25% w/v group (average score: 1.66 ± 0.51) notably decreased the adhesion score compared with the control group (average score: 3.5 ± 0.54) (*p* < 0.05, Figure 3a,b). At the same time, other concentrations (the average score of 0.5% w/v group: 2.16 ± 0.75, and the average score of 1% w/v group: 2.5 ± 1.49) were less effective than the 0.25% group.

**TABLE 3:** Adhesion formation grading based on the Nair et al. scoring system

Rat	Normal	Control	Three concentrations of (SAE)		
			0.25% (w/v)	0.5% (w/v)	1% (w/v)
1	0	3	2	3	4
2	0	4	1	2	2
3	0	4	1	1	1
4	0	3	2	3	3
5	0	3	2	2	2
6	0	4	2	2	3

### 3.3 The effect of SAE on the inflammatory cytokines (IL‐6 and TNF‐α)

**FIGURE 4:**
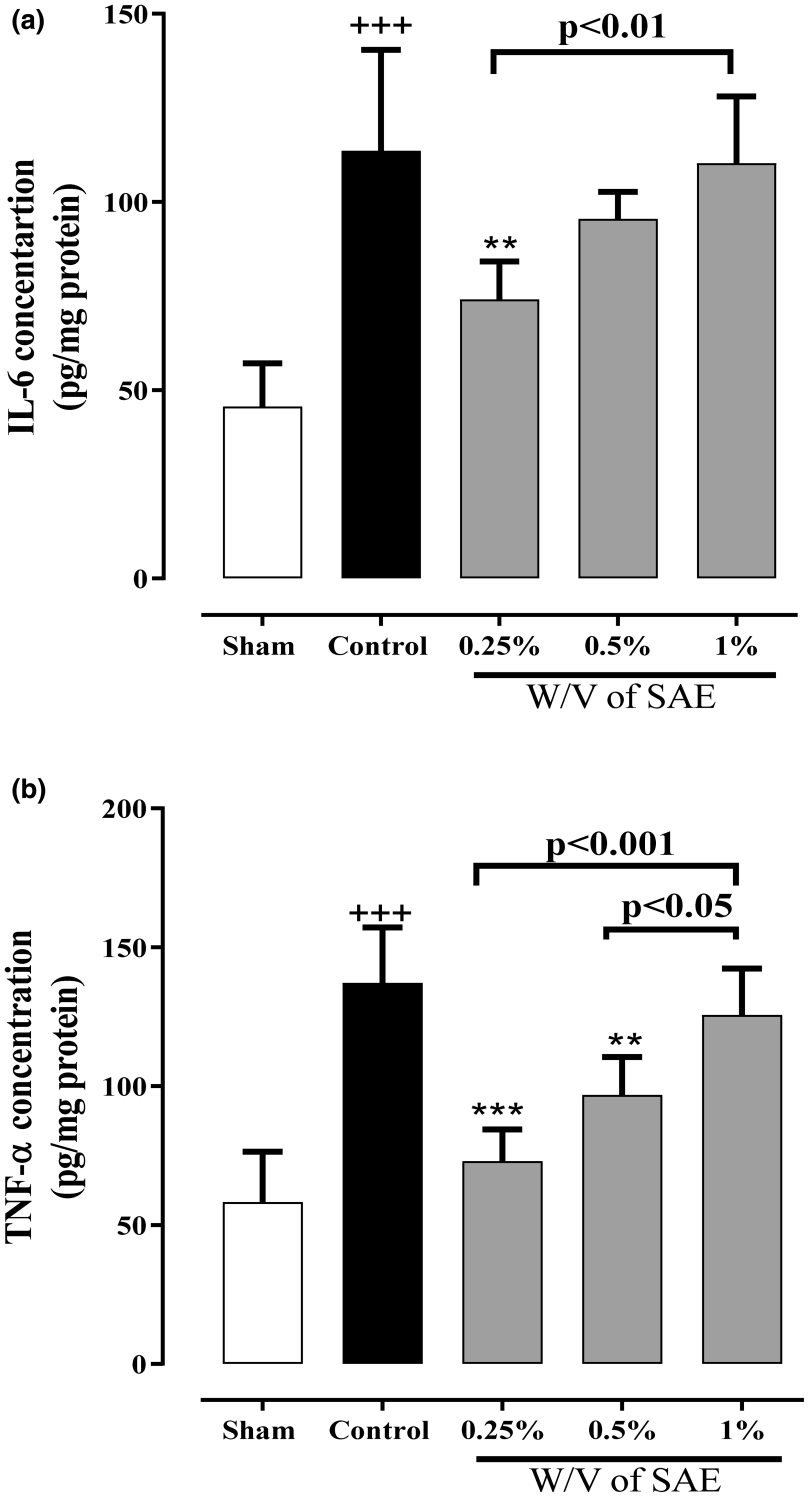
The effect of different concentrations of *S. aromaticum* extract (SAE) on inflammatory biomarkers (a) IL‐6 and (b) TNF‐α levels following post‐operational‐induced peritoneal adhesion. Data were presented as mean ± SD (*n* = 6). ^+++^ *p* < 0.001 compared with the sham group, ***p < 0.001 and **p < 0.01 compared with the control group. The lines illustrated a significant difference between the two groups shown.

Following the PA induction, IL‐6 and TNF‐α levels were significantly augmented in the control group compared to the sham group (*p* < 0.001 for both cases, Figure 4a,b). In contrast, two lower concentrations of SAE (0.25% and 0.5% w/v) diminished inflammatory cytokine levels than the control group. However, the 0.25% w/v SAE group could only significantly reduce the level of IL‐6 compared to the control group (*p* < 0.01, Figure 4a). Furthermore, both the 0.25% and 0.5% w/v of SAE groups meaningfully attenuated the level of TNF‐α in comparison to the control group (*p* < 0.001 and *p* < 0.01, respectively, Figure 4b).

### 3.4 The effect of SAE on the fibrosis (TGF‐β1) and angiogenesis (VEGF) biomarkers

**FIGURE 5:**
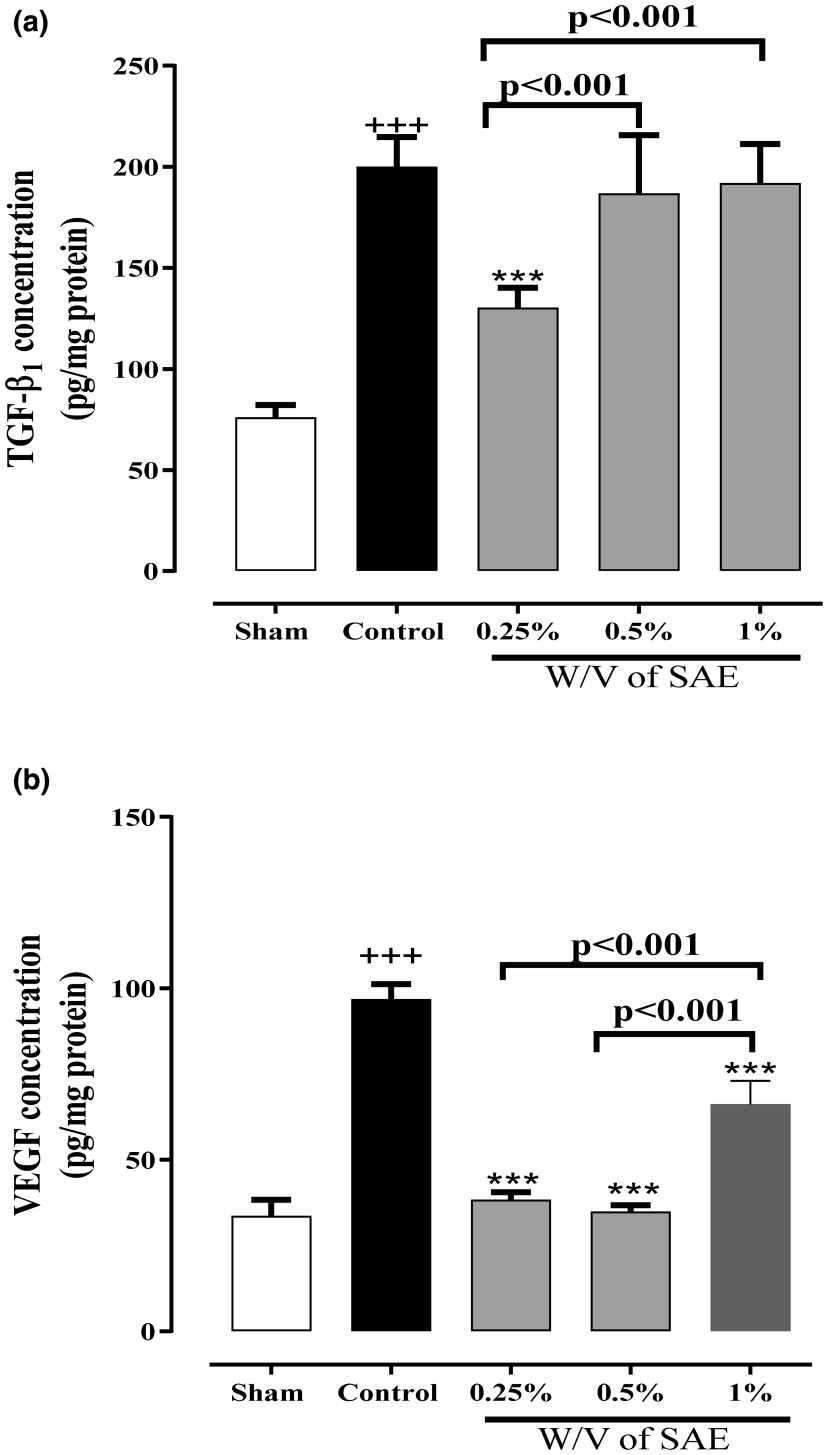
The effect of different concentrations of *S. aromaticum* extract (SAE) on (a) fibrosis factor TGF‐β1 and (b) angiogenesis factor VEGF levels following post‐operational‐induced peritoneal adhesion. Data were presented as mean ± SD (*n* = 6). ^+++^ *p* < 0.001 compared with the sham group and ***p < 0.001 compared with the control group. The lines represent a significant difference between the two groups shown.

In the control group, TGF‐β1 and VEGF levels significantly increased compared with the sham group (*p* < 0.001 for both cases, Figure 5a,b). However, 0.25% w/v of the SAE group remarkably decreased the TGF‐β1 level compared with the control group, and the effect of this group was significantly higher than the 0.5% and 1% w/v of the SAE groups (*p* < 0.001 for all cases, Figure 5a). Intriguingly, all three SAE concentrations remarkably diminished VEGF levels compared with the control group, and the effects of the 0.25% and 0.5% w/v of SAE groups were significantly more substantial than the 1% w/v of the SAE group (*p* < 0.001 for all cases, Figure 5b).

### 3.5 The effect of SAE on the indexes of oxidative stress (NO, MDA and GSH)

**FIGURE 6:**
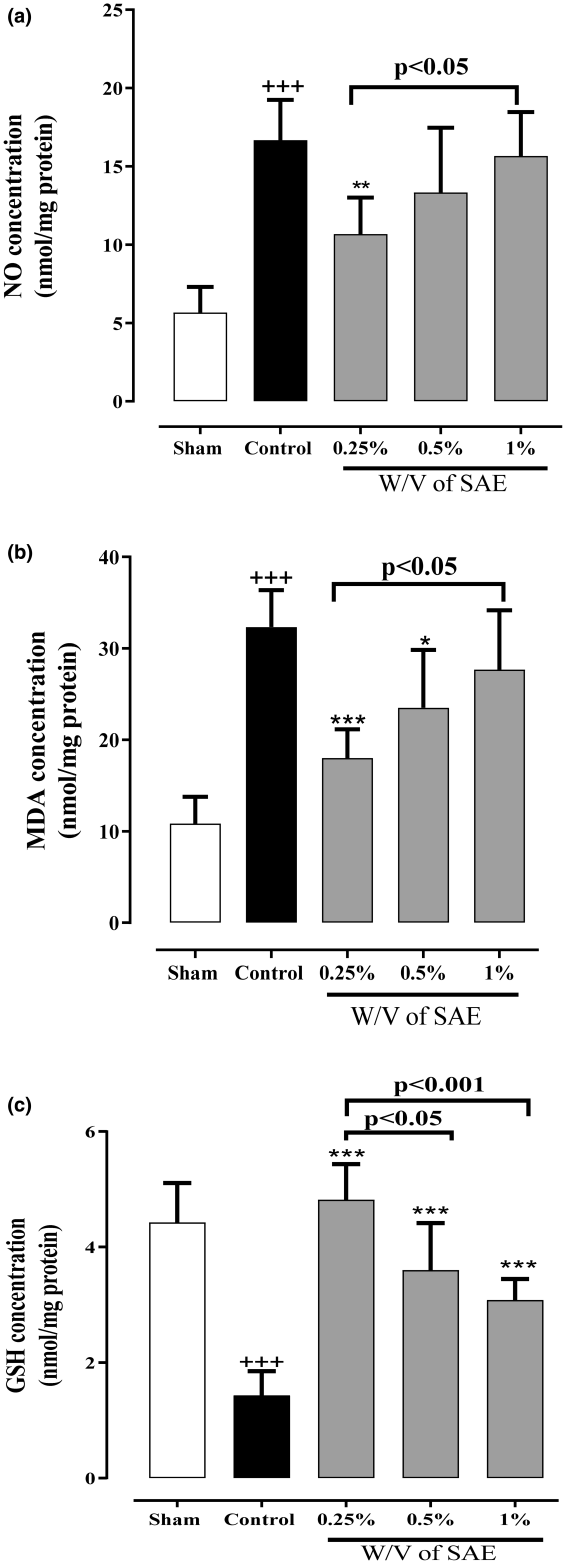
The effect of different concentrations of *S. aromaticum* extract (SAE) on oxidative biomarkers (a) NO and (b) MDA, and anti‐oxidative marker and (c) GSH levels following post‐operational‐induced peritoneal adhesion. Data were presented as mean ± SD (*n* = 6). ^+++^ *p* < 0.001 compared with the sham group, ***p < 0.001, **p < 0.01, and *p < 0.05 compared with the control group. The lines represent a significant difference between the two groups shown.

The control group had meaningfully more NO and MDA levels and fewer GSH levels compared to the sham group (*p* < 0.001 for all cases, Figure 6a–c). On the contrary, all three concentrations of SAE decreased NO and MDA levels and enhanced GSH levels compared with the control group (Figure 6a–c). However, 0.25% w/v of the SAE group remarkably reduced both NO and MDA levels compared with the control group (*p* < 0.01 and *p* < 0.001, respectively, Figure 6a,b), in which the effect of this concentration was significantly higher than the 1% w/v of the SAE group (*p* < 0.05 for both cases, Figure 6a,b). Our results also revealed that the 0.5% w/v of the SAE group significantly abolished MDA levels in comparison to the control group (*p* < 0.05, Figure 6b). Furthermore, all three concentrations of SAE notably enhanced GSH levels compared with the control group (*p* < 0.001 for all cases, Figure 6c). Moreover, the effects of the 0.25% w/v of the SAE group were remarkably more remarkable than the 0.5% and 1% w/v of the SAE groups (*p* < 0.05 and *p* < 0.001, respectively, Figure 6c).

## 4 DISCUSSION

Intra‐abdominal adhesion formation is a major common complication after abdominal surgeries that can cause significant morbidity and increase medical costs. The formation of adhesion bonds is a complex process that involves inflammation, angiogenesis, fibrinolysis, peritoneal tissue repair, and other biochemical events. However, a “gold standard” for PA prevention and treatment has not been determined until now (Herrick et al., 2000; Wei et al., 2017). Cloves are dried flower buds of *Syzygium aromaticum* (L.) Merr. Et Perry and from a long time ago is famous for toothache and mouth and throat inflammation. The most substantial bioactive clove components are eugenol, eugenol acetate, and β‐caryophyllene. *Syzygium aromaticum* extract (SAE) possesses several pharmacological effects, including analgesic, antimicrobial, antitumor, anti‐inflammatory, antioxidant and cytotoxic properties. The potential anti‐inflammatory impacts of clove have also been shown by the inflammatory mediator's inhibitory activity, including IL‐1β, TNF‐α, NO, and IL‐6 (Bhowmik et al., 2012; Dibazar et al., 2015; Rodrigues et al., 2009; Rusmana et al., 2015; Singletary, 2014). Therefore, the current experiments investigated the protective effects of hydroalcoholic extract of *S. aromaticum* (0.25%, 0.5%, and 1% w/v) on the post‐operational‐induced PA formation in a rat model.

For PA induction, we used the scraping model due to its similarity to the adhesion formed by abdominal or pelvic surgeries (Bachiega et al., 2012; Nair et al., 1974). Moreover, we used the Nair et al. (Nair et al., 1974) scoring system to investigate the severity and type of adhesion bonds. Our results showed that the control group's Nair et al. adhesion grade was markedly higher and significantly lower in the 0.25% group.

Considering the formation of PA as an inflammatory process (Sahbaz et al., 2015), we measured the levels of IL‐6 and TNF‐α as inflammatory cytokines in the peritoneal fluid associated with the degree of adhesions present (Cheong, Laird, et al., 2002; Cheong, Shelton, et al., 2002). Our results revealed that the levels of measured inflammatory cytokines in the control group were significantly raised. On the contrary, all three concentrations of SAE reduced IL‐6 and TNF‐α levels. Moreover, the anti‐inflammatory effect of the lowest concentration of SAE (0.25% w/v) was more potent. Previous studies revealed the SAE anti‐inflammatory effect incubated with LPS stimulated murine macrophage on the cytokine production at 100 μg/ml (Bachiega et al., 2012). Furthermore, in vitro study by S. Dibazar et al. showed anti‐inflammatory properties of *S. aromaticum* hydroethanolic extract (0.001–1000 μg/ml) against mouse peritoneal activated macrophages on the production of NO, TNF‐*α*, IL‐6, and IL‐12 levels (Dibazar et al., 2015). Furthermore, it has been investigated that oral administration (for 26 days) of eugenol (33 mg/kg) as the most found component in clove and ginger oil (33 mg/kg) in rats with adjuvant‐induced severe chronic arthritis (by injecting 0.05 ml of a refined suspension of dead *Mycobacterium tuberculosis* bacilli) showed a significant anti‐inflammatory effect of eugenol as the major component of SAE (Sharma et al., 1994). These studies could support our results regarding the anti‐inflammatory effect of SAE.

TGF‐β is a highly potent chemotactic for fibroblasts, macrophages, and other inflammatory cells. It also acts as an intense stimulant for cell proliferation, differentiation, and angiogenesis (Chegini, 1997; Williams et al., 1992). Overexpression of TGF‐β1 is associated with adhesion formation (Williams et al., 1992), possibly through a mechanism involving local regulation of PAI‐1 (Holmdahl et al., 2001). Prevention of TGF‐β production postoperatively may help prevent adhesion formation; for example, using antisense oligonucleotides to TGF‐β is a helpful technique in adhesion prevention (Chegini, 1997). Our results showed that all three concentrations of SAE considerably mitigate the TGF‐β1 level following post‐operational‐induced PA. Moreover, the effect of the lowest concentration of SAE (0.25% w/v) was more significant. The inhibitory effect of SAE on the level of TGF‐β1 has been proved in vitro and in vivo; such as in the study of M. Abdelrahman et al. about the impact of clove extract (200 mg/kg, oral gavage, three times a week for 5 weeks) and honey extract (200 mg/kg, oral gavage, three times a week for 5 weeks) on the inflammatory cytokines and liver function enzymes in experimental rats fed on carbon tetrachloride (0.4 ml, oral gavage, three times a week for 10 weeks) (Abdelrahman et al., 2018).

Vascular endothelial growth factor (VEGF), also known as vascular permeability factor, stimulates angiogenesis, increases vascular permeability, and acts as a principal pro‐angiogenic agent in wound healing and pathogenic processes (Ferrara, 2002). The up‐regulation of VEGF is an essential factor in forming post‐operative intra‐abdominal adhesions (Cahill et al., 2006; Cahill & Redmond, 2008). Saltzman et al. demonstrated that the single IP administration of a neutralizing antiserum to VEGF (1.0 ml) limits post‐operative adhesion formation in mice model of induced PA (Saltzman et al., 1996). Moreover, the study of Ö. Moraloglu et al. demonstrated that utilizing a single dose of Bevacizumab or Avastin® as a monoclonal antibody against VEGF (1 ml of 5 and 7.5 IU) effectively prevented post‐operative PA formation in a rat model (Moraloglu et al., 2011). Our results revealed that all three concentrations of SAE remarkably attenuate VEGF levels following post‐operational‐induced PA. The antiangiogenic effect and inhibition of the sprouting blood vessels of stem and bark extract SA were investigated *in vivo* on rat aortic rings (Aisha et al., 2011).

Nitric oxide metabolites (NO) are a free radical synthesized from L‐arginine by inducible nitric oxide synthase (iNOS) and have a beneficial role in preventing post‐ischemic tissue injuries, including post‐operative PA formation in rats (Özden et al., 1999). MDA, a result of lipid peroxidation, is generated due to the toxic effects of active oxygen radicals, which destroy unsaturated fatty acids in the membranes. GSH level, an essential component of the cellular defense mechanisms against radical‐mediated tissue injury, has been used as an oxidative stress index induced by reactive oxygen species (Ara et al., 2005; Ara et al., 2006). Our results showed that SAE markedly decreases NO and MDA and enhances GSH levels following post‐operational‐induced PA. This finding is consistent with the protective effects of SAE against oxidative damage during PA formation. In previous studies, IP administration of antioxidant agents such as Iranian propolis (200, 100, and 50 mg/kg for 14 continuous days) (Askari et al., 2018) and melatonin (4 mg/kg before closure and for 10 successive days) (Ara et al., 2005) in experimental PA model in rats, significantly decreased NO and MDA and enhanced GSH levels. It has been proved that the hydroethanolic extract of clove represents significant anti‐oxidative activity through the scavenging of different free radicals such as NO and also iron chelation which participates in many pathophysiological conditions of diseases (Kumari et al., 2016). In one study, rats with thioacetamide‐induced cirrhosis (0.03% in drinking water for 16 weeks) showed a progressive increase in lipid peroxidation and a concomitant reduction in GSH levels oxidative stress has been reported (Ali et al., 2014). Contextually, oral administration of eugenol‐rich fraction of *Syzygium aromaticum* (for 9 weeks, 1 week after discontinuation of thioacetamide) significantly increased GSH (Ali et al., 2014). Furthermore, Pretreatment with clove essential oil (0.025, 0.05, and 0.1 ml/kg, IP, for 3 weeks) decreased the oxidative stress assessed by MDA and reduced GSH levels in mice's brain with a scopolamine (0.3 mg/kg, IP)‐induced deficiency in memory and learning as a result of the reduction in the oxidative stress (Halder, Mehta, Kar, et al., 2011). These studies supported the anti‐oxidative effects of SAE, and our results are in line with them.

## 5 CONCLUSION

This study showed that intra‐abdominally administration of SAE significantly reduced the severity of post‐operative PA formation according to alleviating adhesion scores, inflammatory cytokines, oxidative factors, fibrosis, angiogenesis biomarkers, and stimulating anti‐oxidative factors. Therefore, SAE can be considered a potential and cost‐effective agent for preventing post‐operative adhesions. Furthermore, we could suggest the authors to continue the study with its clinical evaluation. However, the detailed mechanism of action and the optimal dose should be further investigated.

## AUTHORS CONTRIBUTIONS

VRA, VBR, and HR proposed the conception; VRA and HR designed and implemented the experiments; EA, HR, MG, MH, and MI participated in animal and in vitro assays; VRA, VBR, and MI participated in the data processing and statistical analysis; EM, VBR, VRA, and MI wrote and revised the article. All authors read and approved the final manuscript.

## FUNDING INFORMATION

This study was financially supported by the research council of Mashhad University of Medical Sciences (Grant number: 970004).

## CONFLICT OF INTEREST

The authors declare that they have no conflict of interest to disclose.

## CONSENT FOR PUBLICATION

All authors read the manuscript and agreed to publish it.

## ETHICS STATEMENT

The ethical committee of Mashhad University of Medical Sciences approved the study protocol by grant number 950312 and the honest number IR.MUMS.MEDICAL.REC.1398.686 (approval date: 2019‐07‐30).
